# Supplementary material for: Immune genes are hotspots of shared positive selection across birds and mammals
Source: eLife. 2019 Jan 8;8:e41815. doi: 10.7554/eLife.41815 (PMC6338464; doi:10.7554/eLife.41815)
Supplement: Supplementary file 1. [file elife-41815-supp1.zip › Shultz_Sackton_Supp_Table_Descriptions_README.pdf]

Supplemental Table 1. List of all species used in study, and data sources for sequences used in gene alignments.

Supplemental Table 2. Raw model results for each gene with the gene tree as input.

Supplemental Table 3. Raw model results for each gene with the species tree as input.

Supplemental Table 4. KEGG pathway enrichment results with chicken as reference organism. High-confidence selection gene set is used as the test set, and all genes tested is used as the gene universe.

Supplemental Table 5. KEGG pathway enrichment results with zebra finch as reference organism. Positively selected genes in birds are used for the test set, and all genes tested are used for the gene universe.

Supplemental Table 6. KEGG pathway enrichment results with human as reference organism. Positively selected genes in birds are used for the test set, and all genes tested are used for the gene universe.

Supplemental Table 6. Gene set enrichment results from Spearman rank correlation ρ values calculated from log-transformed lineage selection values and body mass.

Supplemental Table 7. Median gene lengths for each pathway identified in the KEGG pathway enrichment with chicken as the reference organism. We also report the probability that any gene in a pathway is under selection based on alignment length, the expected number of genes under selection, the observed number of genes under selection, and the results of a Fisher's exact test comparing these two values.

Supplemental Table 8. Gene set enrichment results using the rho values estimated with Spearman's rank correlation values between body mass and PC1 using gene trees as the phylogeny.

Supplemental Table 9. Fisher's exact test results for overlaps between bird and mammal significantly selected genes at four FDR p-value cutoffs for significance.

Supplemental Table 10. Results of logistic regressions for the association between genes under selection in mammals, and genes under selection in birds and alignment length. Results are shown for different FDR-corrected p-value cutoffs of selection.

Supplemental Table 11. Detailed information on all transcriptome datasets used in analyses.

Supplemental Table 12. Logistic regression results for all comparisons between bird differential expression results (up-regulated vs. non-regulated and down-regulated vs. non-regulated) and bird selection results.

Supplemental Table 13. Mann-Whitney U-test results comparing the bird and mammal standardized, absolute mean β values between sets of genes either under selection in both birds and mammals, under selection in birds only, or not under selection.
